# Physical Limits to Auditory Transduction of Hair-Cell Bundles probed by a Biomimetic System

**DOI:** 10.1038/srep11470

**Published:** 2015-06-15

**Authors:** Taegeun Song, Woo Seok Lee, Kang-Hun Ahn

**Affiliations:** 1 Department of Physics, Chungnam National University, Daejeon, 305-764, Republic of Korea; 2 The Abdus Salam International Centre for Theoretical Physics, Strada Costiera 11, 34151 Trieste, Italy

## Abstract

Inspired by auditory hair cells of lower vertebrates, we design and fabricate an opto-electro-mechanical sensor at the border of its spontaneous activity, called Hopf bifurcation critical point. As proposed for biological hair cells, we observe that, as the system approaches the critical point, the frequency selectivity and the force sensitivity are enhanced. However, we find that the enhancement has limits because of its intrinsic nonlinearity, even at the critical point. We also find that the minimally detectable force is not influenced by the active feedback force despite its enhanced sensitivity. This is due to the inevitable heating of the hair bundle, which implies that the active amplification of the hair cell bundle might not lower the threshold level of detectable sound.

Auditory sensory organs are known to have counteracting mechanisms against mechanical friction to select frequency, as T. Gold suggested, “…an electromechanical action takes place whereby a supply of electrical energy is employed to counteract the damping.^1^” It is nowadays widely accepted that the molecular motors in stereocilia of hair cells work against the mechanical friction^2^. There has been an important hypothesis that auditory hair cells of lower vertebrates amplify the movement of their hair bundles^3^ by locating themselves on the border of their spontaneous activity^4^,^5^,^6^. This border is called a Hopf bifurcation critical point which has been extensively studied in nonlinear dynamics^7^.

The Hopf bifurcation hypothesis has attracted great attention because it may explain puzzling aspects of hearing in a very generic model. According to the hypothesis, when the hair bundle is at its critical point, the mechanical displacement sensitivity to stimulus *F* diverges as *F*^−2/3^ 4,5. Furthermore, the feedback action in the hair cell bundle can reduce damping, which sharpens frequency selectivity. In principle, the quality factor Q (peak frequency divided by its line-width) can be up to infinity for weak stimulus limit^4^. In addition to the sharp tuning effects against viscous damping, the hair cell’s amazingly low stimulus threshold is also a striking feature of hearing^8^,^9^.

The purpose of this work is to indirectly investigate the physical limits of the hair cell bundle’s sensitivity, frequency selectivity, and signal-to-noise ratio, when the hair cells are at the critical point. We use a system which mimics hair-cell bundles and is easily controllable through minute changes in electronic resistance.

While noise and dissipation play important roles in biomechanical systems, their strength and functional dependence are difficult to estimate when the system is in non-equilibrium. The precise modeling of noise and dissipation is critical, especially when the system is near the Hopf bifurcation criticality. Thus, this biomimetic study on the Hopf bifurcation hypothesis is more than numerical simulations with many assumptions.

We design and fabricate a biomimetic resonator where its dissipation is effectively controlled through a parameter. We find that, beyond a certain threshold, the sensitivity is a nonlinear function of −2/3 law of input stimulus, which is a typical sign of Hopf bifurcation. Below the threshold stimulus, the mechanical response is a linear function of input stimulus. In this linear region, the frequency selectivity as well as sensitivity can be greatly enhanced as the system approaches the critical point, which is in line with Gold’s suggestion^1^. However, we find that, due to the nonlinear nature of the critical hair cells, the enhancement of sensitivity and frequency selectivity are intrinsically limited. Thermal noise plays less of a role when compared to the nonlinearity in this case. It is also demonstrated that the enhanced sensitivity does not lead to lowering the threshold stimulus. We find that, regardless of how close the system is to the critical point, the minimal detectable stimulus is not meaningfully changed. This is because the reduction of the dissipation near the critical point inevitably increases mechanical fluctuation, which has an negative effect on precise measurement.

## The critical hair-cell bundle model

The active feedback action in the hair cell bundle is able to propel oscillations of the artificial hair cell by producing a negative slope in the force-velocity relation. The displacement of the bundle X and its molecular motor’s position *X*_*a*_ follows the equations^5^ : λ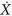 = −*k*_*gs*_(*X* − *X*_*a*_ − *DP*_*o*_) − *k*_*sp*_*X* + *F*(*t*) and λ*_a_*
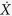
_*a*_ = *k*_*gs*_(*X* − *X*_*a*_ − *DP*_*o*_) + *F*_*a*_. Here *k*_*sp*_ and *k*_*gs*_ are the intrinsic stiffness of the pivot spring and gating spring of the hair bundle, respectively. F(t) is the external force stimulus. λ and λ_*a*_ are the friction of a hair bundle and the friction of adaptation motors, respectively. *F*_*a*_ is the active force deployed by molecular motors which depends on *Ca*^2+^ concentration in the stereocilia. The motors tend to climb up the stereocilia, 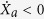, which increases the extension of the gating spring and opens the transduction channels. The tension is released by the influx of *C*_*a*_^2+ ^through the transduction channels. *D* is the gating spring elongation and *P*_*o*_ is the probability for the transduction ion channel to be open. Since the mechanical energy stored in the gating springs is 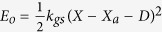 
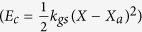
 when the channel is open (closed), the probability for the hair transduction channel to be open 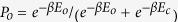 is given by


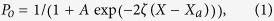


where *β* = 1/*k*_*B*_*T* is the reciprocal of the temperature, A is a constant associated with the intrinsic free energy difference between the open and closed states and *ζ* = 2 *k*_*gs*_*D*/*k*_*B*_*T* is the inverse of a typical length associated with the channel gating which depends on temperature T.

## Method

**Figure 1:**
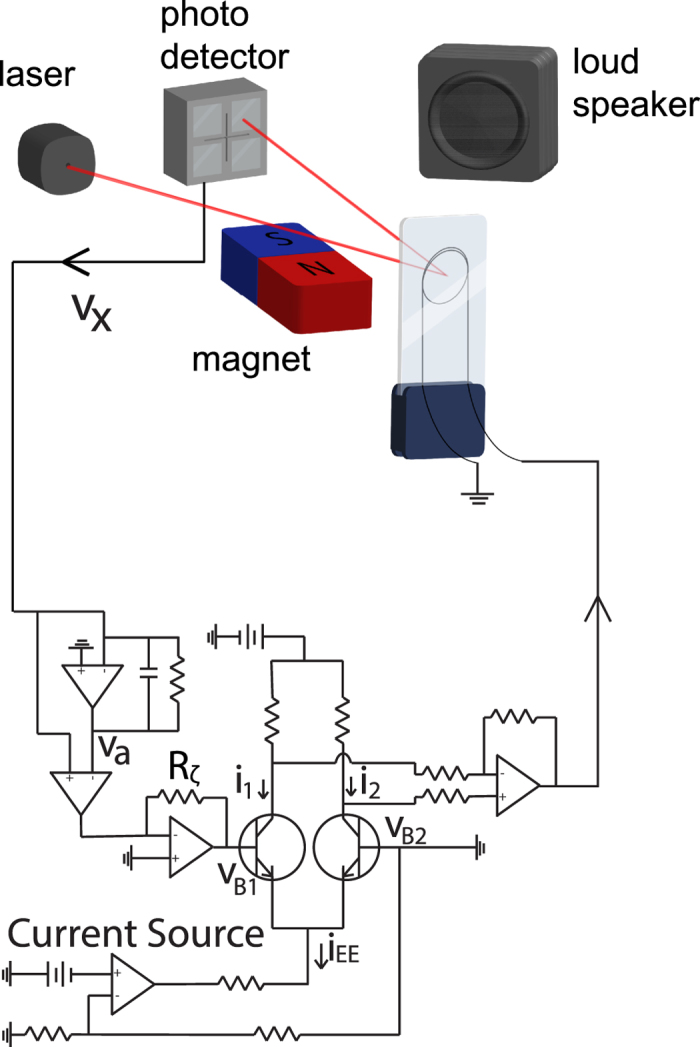
A schematic figure for the experimental setup.

A sketch of our force sensor is shown in Fig. 1. A cantilever is made of a copper loop laminated with polyester sheets^10^. This cantilever is placed in a static magnetic field and subjected to a time dependent Lorentz force when a.c. currents are injected through the loop^10^. A He-Ne laser beam reflects on the tip of the cantilever. The deflection of the reflected beam is measured by a quadrant photodetector and recorded as a voltage *V*_*X*_(*t*). The displacement of the bundle X is proportional to *V*_*X*_ through *V*_*X*_ = *α*_*g*_*X* where *α*_*g*_(=0.032 V/*μ*m) is a proportionality constant depending on the geometry of the setup.

We design the active force in the form of a hyperbolic tangent function using the feedback circuit shown in Fig. 1. *X*_*a*_ is defined to be *X*_*a*_ = *V*_*a*_/*α*_*g*_, where *V*_*a*_ is the output voltage of the leaky integrator in Fig. 1. Thus *X*_*a*_ satisfies


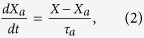


where *τ*_*a*_ is the adaptation time constant which is realized by the RC time constant of the circuit. In this circuit, the current difference is given by 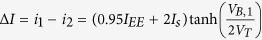 (*V*_*T*_ = *k*_*B*_*T*/*e* is the thermal voltage) which gives the feedback force *F*_*a*_ on the cantilever in a magnetic field.

This force is in a hyperbolic tangential function of the bundle’s deflection Δ*I* ∝ tanh [*ζ*(*X* − *X*_*a*_)] as in the biological hair cell bundle. The parameter *ζ* is controlled by the variable resistance *R*_*ζ*_[*k*Ω] = 2*ζV*_*T*_/*α*_*g*_ The Newtonian equation of motion for the deflection X of the cantilever with an effective mass m is then given by


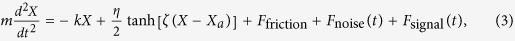


where *k* is the spring constant of the cantilever and *η* describes the strength of the feedback force. In our previous study on a similar biomimetic system^10^, the subcritical Hopf bifurcation could not be achieved, because its 1/*ζ* was fixed to be zero. *F*_friction_, *F*_noise_, and *F*_signal_ are the forces from friction, noise, and sound signal, respectively. We found the following parameters are well fit to our system: *m* = 1 *μ*g, 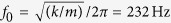, and *τ*_*a*_ = 2 ms.

**Figure 2:**
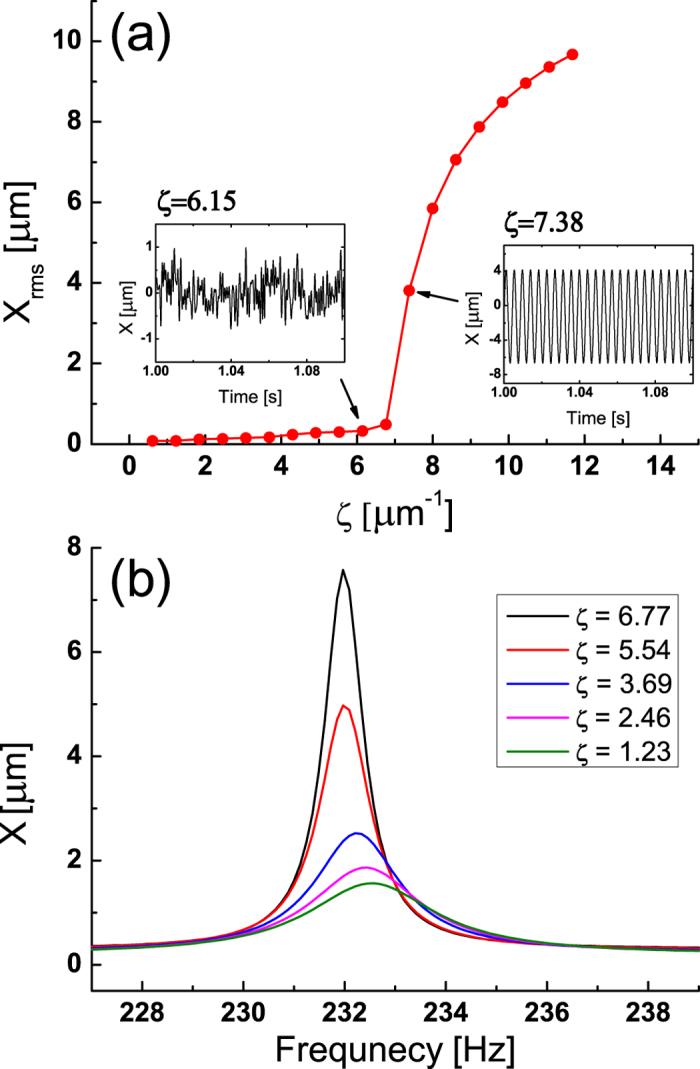
The electro-opto-mechanical system shows the Hopf bifurcation criticality and enhancement of the frequency selectivity. **a**) The root-mean-square value of the displacement of the cantilever as a function of the parameter *ζ* which shows Hopf bifurcation criticality. (**b**) The response of the displacement to a single tone sound as a function of the applied sound frequency for various bifurcation parameter *η*.

In our biomimetic experiment, we used a long-time average for our lock-in amplifier (30 seconds for Fig. 2 and 5 minutes for Fig. 4 and Fig. 6). By using a long-time average, we could more precisely determine the critical point. This was possible because our system is relatively stable compared to biological hair bundles. For experiments using biological hair cells which are less stable, the average time is usually much shorter (2 seconds for Ref. 3). The resulting data in this case would show more fluctuation but the same limitation we discussed would be there.

## Results and Discussion

### Limit to Frequency Selectivity

In Fig. 2 we show our experimental results where we find the Hopf bifurcation criticality using the parameter of *ζ*. The remarkable tunability of our opto-mechanical systems allows us to locate the critical point precisely. As the system approaches the critical point, the frequency selectivity increases. The quality factor Q increases to 250 as *ζ* approaches its critical value 6.77 *μ*m^−1^ (Fig. 2b). Here, the quality factor Q is defined as *f*_*0*_*/*Δ*f*, where *f*_0_ is the peak frequency and Δ*f* is the full width at half maximum (FWHM). This enhanced Q phenomenon has been observed in bullfrog hair cells^3^.

Now let us consider the dynamics of the system near the critical point. Let *X*(*t*) = *X*_*0*_(*ω*)*e*^iω*t*^ + *c*.*c*, *X*_*a*_(*t*) = *X*_*a0*_(*ω*)*e*^iω*t*^ + *c*.*c*, and *F*_*sig*_(*t*) = *F*_*0*_(*ω*)*e*^iω*t*^ + *c*.*c*. We assume that the system is close to the Hopf bifurcation so that these amplitudes are small enough to ensure the approximation of 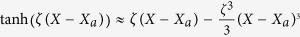. From the comparison of the terms containing *e*^*iωt*^, we get


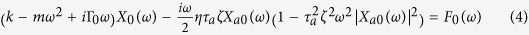



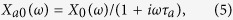


where we assume the frictional force is linear in proportion to the velocity with a frictional constant 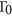; 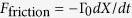. For 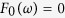, we rewrite equation (4) and equation (5) in terms of *X*_*0*_(ω), i.e.


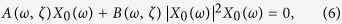


with certain coefficients *A*(*ω*, *ζ*) and *B*(*ω*,* ζ*). The spontaneous oscillation amplitude is written as 
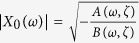
, which must be zero at the critical point. Thus the condition for the Hopf bifurcation criticality is *A*(*ω*_*c*_, *ζ*_*c*_) = 0, which gives the critical values for the control parameter and its oscillating frequency;


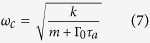



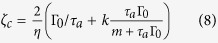


In a linear approximation where only first order terms of 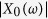 are taken in equation (4) and equation (5), the sensitivity can be written as


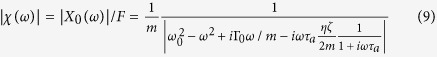


By calculating the FWHM Δ*f* of the linear sensitivity, we can approximate the quality factor as


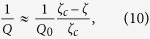


where *Q*_0_ is the quality factor of the cantilever in the absence of the feedback action.

**Figure 3:**
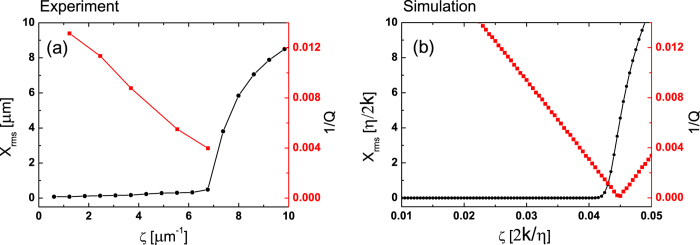
(**a**) Experimental values for the inverse of the quality factor *Q* and the root-mean-square value *X*_rms_ of the displacement as a function of the control parameter *ζ*. (**b**) Theoretical simulation for 1/*Q* and *X*_rms_ when *γ*_0_/*ω*_0_ = 1./(20*π*) and *ω*_0_*τ*_*a*_ = 0.8*π*.

In Fig. 3, we show that the afore mentioned theoretical critical point is shifted from the experimental critical point. In Fig. 3a, our experimental results show the Q factor as the system approaches the critical point, which is defined as a point where spontaneous oscillation suddenly arises. In contrast to the theoretical prediction in equation (10) where Q is infinite at the critical point, we clearly see that the quality factor Q is a finite number which does not exceed 300. *Where does this limitation come from?* Note that the formulae for the criticality in equation (7) equation (8) are obtained based on the ideal assumptions of noise-free and linear response. However, we find the linear approximation leads to an overestimation of the value of the critical point. In Fig. 3b, we plot the amplitude of spontaneous oscillation as a function of the bifurcation parameter *ζ*, and compared it with the value of 1/*Q* which is obtained from the linear response formula of equation (9). In the linear approximation, 1/*Q* becomes zero at the critical point, but we find that it is located inside the region where the spontaneous oscillation amplitude is finite. This discrepancy comes from the fact that the nonlinear terms in equation (6) are limited up to the third order of *X*_0_. If the critical point were obtained from equations including the 5th order of *X*_0_, the critical point would be different from the point shown in equation (7) and equation (8). We note that only the terms up to the third order were used in the analyses of the generic model of Hopf bifurcation^6^ and the hair bundle model in Ref. 4. The spontaneous oscillation amplitude in Fig. 3b was obtained from a numerical simulation ignoring any noise effects, including any higher order terms, thus the different estimation of the critical point is due to the higher nonlinearity over the third order. *The frequency selectivity is limited due to higher nonlinear effects over the third order near the critical point and its Q does not go to infinite unlike the linear case.*

The value of Q for cochlea were found to be 60–250 in the frequency range 1–10 kHz^1^. This value is 15–25 times higher when compared to the value of Q of strings immersed in water^1^. Our biomimetic system shows just about 3 times enhancement of Q by approaching its criticality. As we have shown in this work, this limitation of the enhancement of Q is due to the nonlinearity of the system. This consideration leads us to guess that the Hopf bifurcation mechanism is not enough for counteracting friction in the cochlea.

### Limit to Sensitivity

According to other previous analyses on hair bundles at criticality^4^,^6^, the linear term of 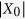 vanishes at the critical point, and the leading term for small amplitude is 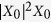, so that 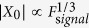. It seems that the sensitivity may diverge to infinity at the critical point. A different type of sensitivity enhancement at a special point can be found in another system^13^. In the previous section, we notice that the critical point (from the linear analysis) is located inside the spontaneous oscillating regime, so that we can predict in this section that the infinite sensitivity is not reached in the subcritical region.

**Figure 4:**
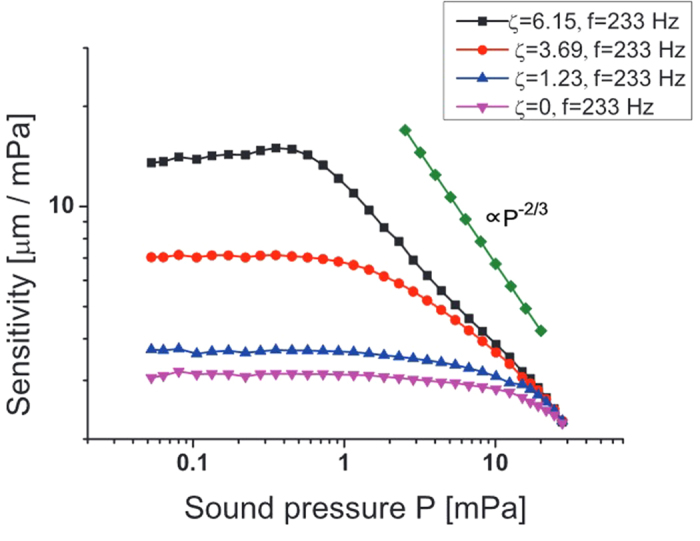
The sensitivity as a function of the pure-tone sound pressure amplitude for various control parameter *ζ*. The frequency of the applied signal is 233 Hz.

In our experiments where sensitivity is measured for various sound pressure levels, the results consistently showed saturation of the sensitivity (Fig. 4). We applied a sound signal to the cantilever where the sound pressure was proportional to the applied signal force *F*_signal_ ∝ *P*, where *P* is the sound pressure applied to the cantilever. The sound pressure was measured using a microphone (Knowles Electronics, NR-23158-000). As shown in Fig. 4, we found the power law behavior of the sensitivity (displacement amplitude divided by sound pressure amplitude) to be close to the −2/3 law. We find that the constant sensitivity (or linear response) always exists below the region of the power-law region.

Unlike a passive and linear resonator, the hair bundle in the linear-response region is not a passive system, but has active feedback control which counteracts friction. In this region, the sensitivity is a function of the control parameter *ζ*. As it approach the critical point, the sensitivity increases (but does not diverge). When the system is close to the critical point, but the displacement amplitude is small enough to ensure the linear response, the sensitivity *χ*(*ω*) = *X*_0_(ω)/*F* can be approximated as


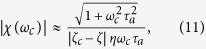


where *ζ*_*c*_ is the parameter value for the critical point from the linear analysis (Equation (8)). This linear theoretical sensitivity seems to diverge at *ζ* = *ζ*_*c*_, but it does not because this ‘linear’ critical point is not reached as it is located inside the spontaneously oscillating region.

### Limit to Minimal Threshold Stimulus

The minimally detectable stimulus threshold is determined by the signal-to-noise ratio of the output. To investigate these quantities, let us consider a spectral density which is defined by;


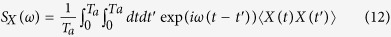


The minimal force which might be detectable can be estimated as


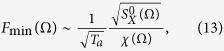


where *T*_*a*_ is the time duration for the pure-tone signal (at angular frequency Ω) and 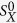 should be understood as *S*_*X*_ in the absence of any sound signals. This relation is estimated from the condition that signal-to-noise ratio (SNR) ≈ 1, where


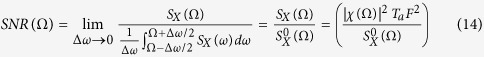


**Figure 5:**
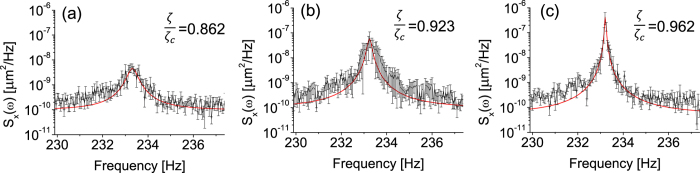
Power spectral density of the artificial hair-cell bundle in the absence of signal for various *ζ*/*ζ*_*c*_.

**Figure 6:**
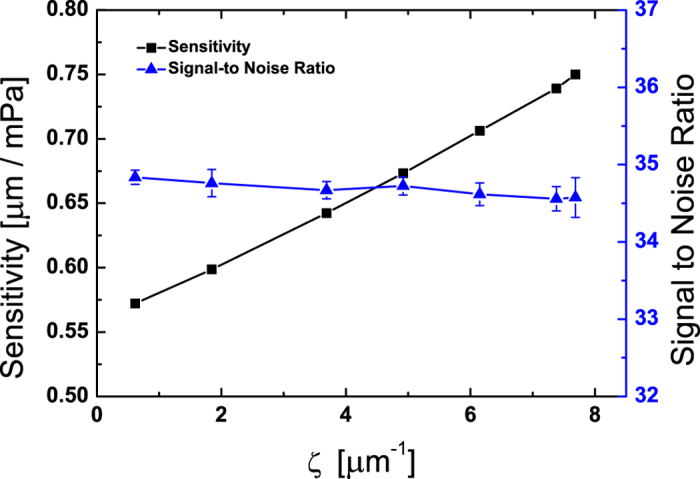
Sensitivity(black square) and Signal-to-Noise ratio(blue triangle). The sensitivity and the signal-to-noise ratio are measured at 0.17 mPa and 231 Hz (the peak frequency is 233 Hz, *ζ*_*c*_ = 7.99).

According to equation (11), the sensitivity increases as 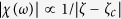, which might indicate the signal threshold might be lowest near the critical point. However, the noise is also enhanced near the critical point. As shown in Fig. 5, the spectral density in the absence of signals increases as the system approaches the critical point. The enhancement of the noise power is an inevitable heating effect which arises from the reduction of the dissipation^11^,^12^. The effective mode temperature of the hair-cell bundle is defined to be 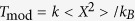 following the equipartition theorem, where 
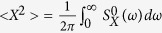
. If we are within the linear approximation, the sensitivity and spectral density are scaled properly so that SNR and *F*_min_ do not depend on the bifurcation parameter *ζ*. In our experiment (Fig. 5), we find that the SNR is basically unaffected by the bifurcation parameter *ζ*, while the sensitivity is sensitively significantly increases as *ζ* approaches *ζ*_*c*_. Therefore, the minimal detectable force in equation (13) does not depend on *ζ*.

## Conclusion

In summary, we designed and fabricated a Hopf bifurcation oscillator in an opto-electro-mechanical system which mimicked auditory hair cell bundles of lower vertebrates. We demonstrated the Hopf bifurcation criticality and showed that the dissipation of the system is significantly changed as we control the bifurcation parameter. The quality factor of the mechanical resonator significantly changed as a linear function of an electrical resistance parameter. The mechanical response shows roughly the −2/3 law behavior as a function of the applied sound pressure signal amplitude, which is a typical characteristic of Hopf bifurcation oscillator. However, we found that the infinite sensitivity is not reached as an intrinsic property of the system. The frequency selectivity was also found to be limited at the critical point.

We found that the infinite sensitivity and frequency selectivity arose only when the hair-cell bundles’ motion was described by mathematical models which contain up to third-order terms (which were discussed in existing theories^4^,^6^). The critical point, which is predicted by this limited nonlinear model, is different from a true critical point in a physical model. These sensitivity and frequency selectivity limitations are due to the fact that the ideal critical point from the limited nonlinear bundle model is actually located inside the true spontaneously oscillating region. At a real critical point, the sensitivity and the frequency selectivity are not divergent. This limitation remains regardless of any cooling of the system.

We showed that the enhancement of the sensitivity near the critical point does not ensure lowering of threshold stimulus. This is because while the reduction of dissipation near the critical point helps to increase the frequency selectivity, but it also enhances the mechanical fluctuation, which is harmful to precise measurement. The minimal detectable force, therefore, does not show any meaningful change near the critical point in spite of its enhanced sensitivity.

## Additional Information

**How to cite this article**: Song, T. *et al.* Physical Limits to Auditory Transduction of Hair-Cell Bundles probed by a Biomimetic System. *Sci. Rep.* **5**, 11470; doi: 10.1038/srep11470 (2015).
